# Opt-in HIV testing in construction workplaces: an exploration of its suitability, using the socioecological framework

**DOI:** 10.1186/s12889-022-13787-5

**Published:** 2022-07-23

**Authors:** Sarah Somerset, Wendy Jones, Catrin Evans, Cecilia Cirelli, Douglas Mbang, Holly Blake

**Affiliations:** 1 School of Medicine, University of Nottingham, Nottingham, UK; 2 NIHR Nottingham Biomedical Research Centre, Nottingham, UK; 3 School of Health Sciences, University of Nottingham, Nottingham, UK

**Keywords:** Workplace, Construction, Men’s health, HIV, Sexual health, Health screening, Health protection, Health promotion

## Abstract

**Background:**

Late diagnosis of HIV remains a challenge, despite improved testing and treatment. Testing is often targeted at high-risk groups; workplace events might normalise testing and allow access to a wider population. The construction workforce has a number of risk factors for HIV. In the Test@Work study, HIV tests were delivered within general health checks to construction employees, with high uptake and acceptability. This paper reports on the experiences of construction managers and health professionals involved in Test@Work and explores the suitability of construction worksites as a venue for opt-in HIV testing.

**Methods:**

Qualitative interviews (*n* = 24) were conducted with construction managers who had facilitated health check/HIV testing (*n* = 13), and delivery partners (*n* = 11) including i) healthcare volunteers who had delivered general health checks (*n* = 7) and, ii) HIV professionals who had conducted HIV testing (*n* = 4) at 21 Test@Work events held on construction sites. Interviews explored their experiences of these events and views towards HIV testing in the workplace. Exit questionnaires (*n* = 107) were completed by delivery partners after every event, providing qualitative data identifying facilitators and barriers to effective delivery. Thematic analysis identified themes that were mapped against a socioecological framework.

**Results:**

Delivery partners reported high engagement of construction workers with workplace HIV testing, peer-to-peer encouragement for uptake, and value for accessibility of onsite testing. HIV professionals valued the opportunity to reach an untested population, many of whom had a poor understanding of their exposure to HIV risk. Managers valued the opportunity to offer workplace health checks to employees but some identified challenges with event planning, or provision of private facilities.

**Conclusions:**

The construction sector is complex with a largely male workforce. Providing worksite HIV testing and education to an untested population who have poor knowledge about HIV risk helped to normalise testing, encourage uptake and reduce HIV-related stigma. However, there are practical barriers to testing in the construction environment. Rapid testing may not be the most suitable approach given the challenges of maintaining confidentiality on construction worksites and alternatives should be explored.

**Supplementary Information:**

The online version contains supplementary material available at 10.1186/s12889-022-13787-5.

## Background

Despite improvements in HIV (human immunodeficiency virus) testing and treatment in recent years, late diagnosis remains a challenge. For example, individuals within Europe who contract HIV typically experience a three-year delay before diagnosis, with many opportunities for testing being missed in the intervening time [1]. In the UK, men who are heterosexual and those aged fifty or older are at particular risk of late diagnosis, as are black African men [2]. Late diagnosis increases the risk of poor health, premature death, and of spreading the disease to others prior to diagnosis and treatment. Improved interventions are therefore needed if the United Kingdom (UK) government is to achieve its goal of ending HIV transmission by 2030 [2].

Traditionally, HIV testing has focused on those believed to be at the highest risk, for example by testing in sexual health clinics. In recent years, there have been developments in community-based testing programmes, but these often still target specific high-risk groups, with events organised at gay pride events, in saunas and in centres for drug users and the homeless [3, 4]. Community testing has been successful at reaching previously untested individuals, and at diagnosing new cases, and has high acceptability. However, targeted testing will miss those who do not fall into these high-risk groups. Further, some who are at high risk for HIV infection may not present themselves for testing due to fear of the consequences of a positive result [5], fear of the stigma associated with testing itself (due to associations with homosexuality or promiscuity [5, 6]), or conflicts between testing and personal perceptions of masculinity [7].

An alternative to targeted testing is to normalise HIV tests via presentation as a routine part of healthcare. This has been advocated by the European Union [8]. Examples of this strategy have included offering testing to all new registrants at general practices [9]; testing at outpatient departments and in Emergency departments [10]; and offering testing routinely to all women attending for maternity care, which has been standard practice in most European countries since the early 2000s [11]. These approaches have been successful, with a high acceptability to patients, good uptake and identification of new cases of HIV [9–11].

Testing which is normalised in this way is offered regardless of perceived risk. This avoids individuals feeling that they are being targeted or judged for their lifestyle [12]. It is also important given that many underestimate their risk of being infected. Those who perceive HIV as a disease which affects certain stereotypical groups are less likely to undertake testing [13, 14]. A recent study of students in Poland found that they associated HIV with homosexuality, drug use and sex work and therefore would not seek testing despite undertaking risky activities [15]. Other reported examples of poor risk perception were identified in a cohort of Africans in London expressing surprise at testing positive, as they had failed to recognise that they were at risk, despite engaging in risky behaviours [16]; and evidence that many pregnant women, have insufficient understanding to assess their risk accurately [17].

There are challenges to normalising HIV testing in regular healthcare settings. One barrier to such testing being more widespread and successful is the expertise and beliefs of health professionals, who underestimate the acceptability to their patients, as well as overestimating the time taken to provide testing and lacking knowledge about how testing might be conducted [5, 18]. It can also be difficult to fit HIV testing in alongside other demands in a busy healthcare environment. Additionally, testing lower risk populations will identify fewer cases, and therefore is proportionately more expensive [19].

An alternative route to providing widespread, normalised HIV testing is through the workplace. This provides access to those who may not regularly attend healthcare facilities due to being younger [20] and in good health; or who lack time or opportunity to attend health services due to work commitments [21, 22]. Targeted interventions in male-dominated workplaces can provide an effective way to access these under-served populations [23]. The workplace has been used as a venue for HIV testing in high prevalence regions such as Sub-Saharan Africa, as the convenience and accessibility of testing improves uptake [24]. Such testing is particularly helpful for working populations who may be at increased HIV risk due to their lifestyle, such as the construction workforce who often live away from home, drink alcohol to excess and may take illegal drugs [25, 26].

The research described in this paper is part of the Test@Work programme [27–31], which was undertaken to evaluate the provision of HIV testing embedded within a general health check in UK construction worksites. Construction workforces are often transient [32], have poor worker health [33–35] and may have workers with high-risk lifestyles [36–38]. Test@Work found a high uptake of HIV testing in this context; 97% of health check participants opted to have a consultation about sexual health and 82% had an optional HIV test, of whom 78% had not previously been tested [27, 31]. There was good acceptability with the workforce [28]. The current study examines the perspectives of the managers who had arranged for testing to take place and the health professionals who provided the general health checks and HIV testing. The aim of this research is to explore the suitability of the construction workplace as a location for HIV testing, from the perspective of construction organisations, and delivery partners.

**Fig. 1:**
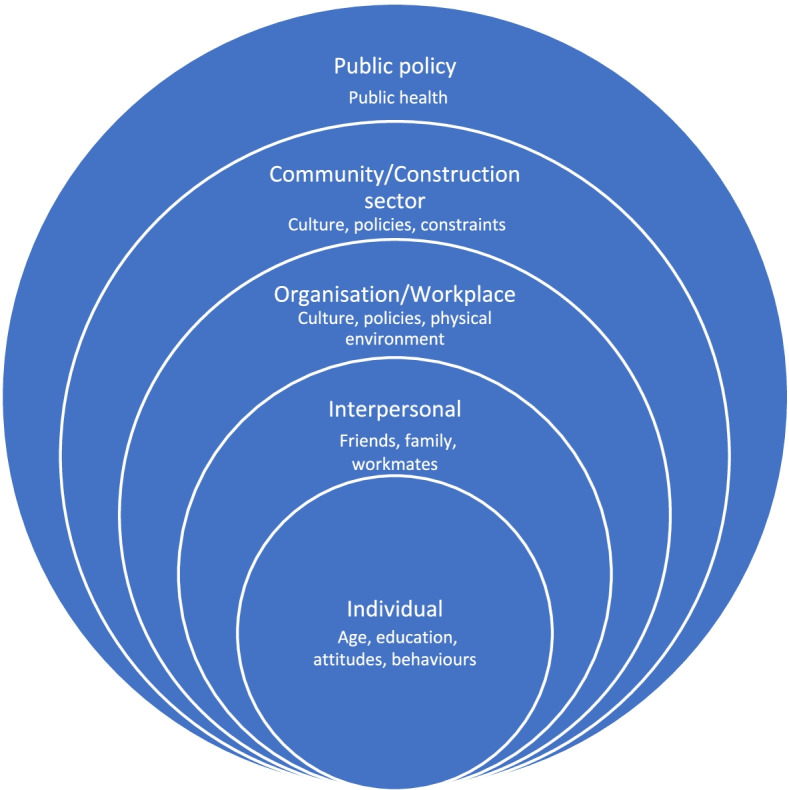
The five levels of the socio-ecological framework

This research has been undertaken within the context of a socio-ecological framework (SEF). In a SEF, it is recognised that personal and environmental factors and the interactions between these are important in shaping behaviours [39, 40]. SEFs have been used in a range of contexts: by the Centers for Disease Control and Prevention (CDC, the national public health agency of the United States) and the World Health Organization (WHO, a specialised agency of the United Nations responsible for international public health) to underpin approaches to tackling violence [41]; as a tool to assess barriers to HIV care in an at-risk population [42]; to model HIV risk and identify research priorities [43]; and in workplace contexts to explore the effectiveness of safety or health interventions [44, 45]. In this research, SEF is used to explore how the lived experiences of those within the construction industry link to health behaviour and health outcomes in relation to HIV. The SEF comprises five levels as defined by McLeroy [46] and Golden [47] (intrapersonal/individual, interpersonal, organisation, community and public policy), summarised in Fig. 1. Following Lingard et al. [45], construction workplaces are defined as ‘organisations’ and the wider construction industry as ‘community’.

## Methods

### Research setting and intervention

This research was conducted alongside the Test@Work programme, which is described in detail elsewhere [27–31]. Health checks (*n* = 426) including optional HIV testing (*n* = 348 opted in) were provided to workers at 21 events on construction sites in the UK. Those attending for health checks were predominantly male (81.7%), white (88%) and heterosexual (97.4%), reflecting the characteristics of the wider construction workforce in the UK. Most were UK-born (87.3%) with 5% self-reporting as being European and 6.2% from other parts of the world. In terms of age, they were fairly evenly spread, with just a slight over-representation of those aged 31–40 (28.6%).

**Fig. 2:**
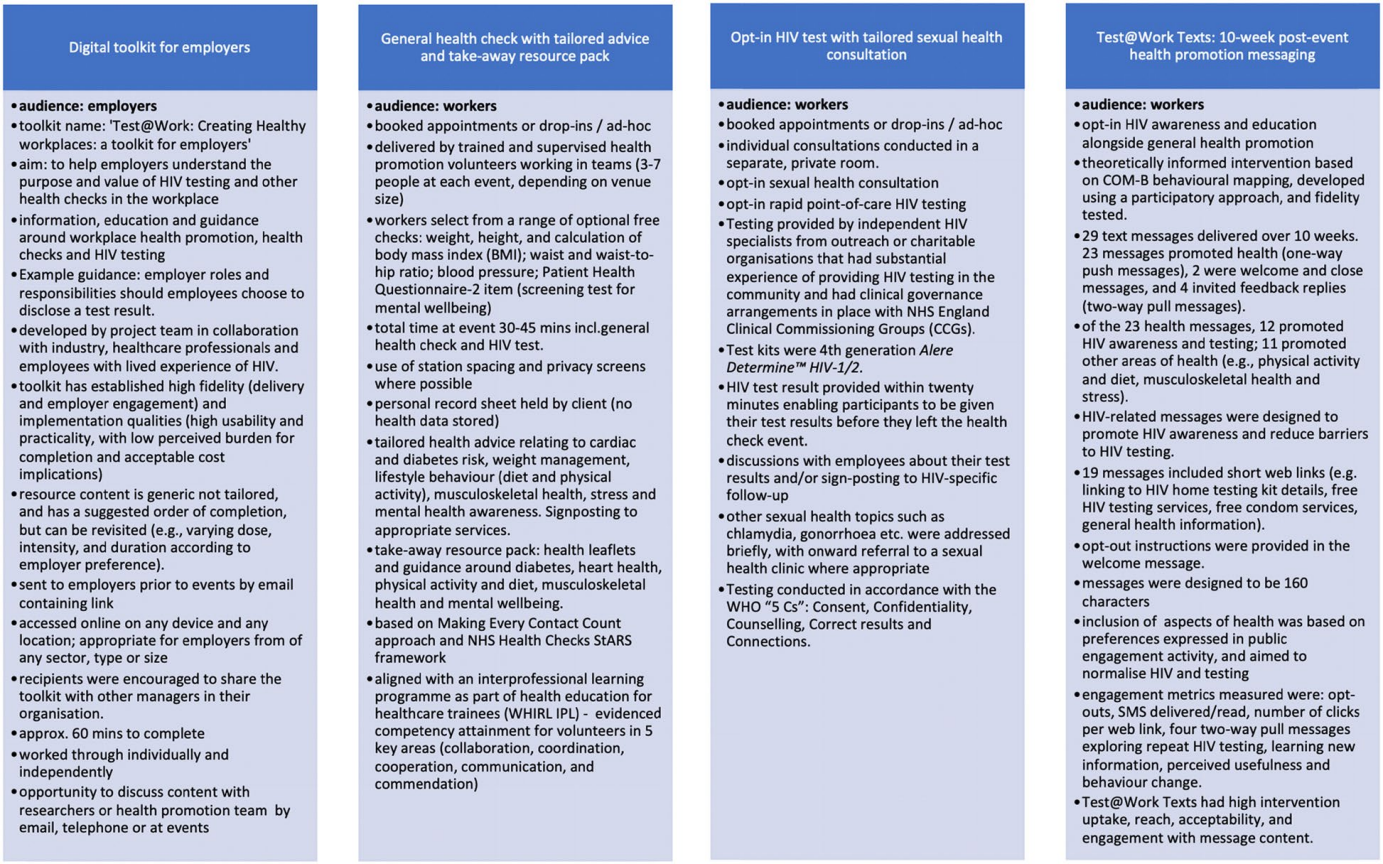
Test@Work intervention

The health checks included confidential measures of weight, height, and calculation of body mass index (BMI), waist and waist-to-hip ratio measurements, blood pressure, a screening test for mental wellbeing and opt-in sexual health consultation with rapid, point-of-care HIV testing. All participating workers received a record of their test results, tailored health advice and signposting, and a take-away pack of resources with health leaflets and guidance around diabetes, heart health, physical activity and diet, musculoskeletal health and mental wellbeing [27]. Those who had taken part in a health check were also invited to participate in an additional post-event health promotion intervention called Test@Work Texts [30], consisting of a series of text messages with HIV awareness and education, together with general health promotion, delivered over 10 weeks. The details of each component of Test@Work are shown in Fig. 2.

Events were organised at 10 companies through key contacts (e.g., site managers or health and safety managers) who had responded to an invitation (phone/email) from the research team. Testing was offered to all employees on site, on the day of testing. All workers based on the host sites were informed about the health checks and what they entailed, by site managers. On the day of the health check event, all workers who expressed an interest in taking part were provided with an explanation of each health check that was available, by a project researcher, and the estimated time to undertake test, receive results and tailored advice. Workers could opt into all, or some of the checks at their own discretion (ie. they were at liberty to engage in selected tests, and decline others without needing to provide any explanation, including the HIV test). Although opt-out approaches have been found to be acceptable in clinical settings (e.g., primary care), it has been recognised that any pressure to test is likely to be poorly received [12]. Further, workers may have had concerns (albeit inaccurate) about medical data being shared with employers, line managers or colleagues and this may be a disincentive to test. Our opt-in approach was therefore deemed to be more suitable for this workplace setting. Results of the general health checks were recorded on a sheet that was handed to the worker - the research team did not store test results. Although the logistics varied from site to site depending on space availability, the general health checks (non-HIV element) were conducted in one area with the volunteer team, and the HIV testing was conducted in a separate private area. Each client met individually with the HIV providers and results were given in a private space, by two service providers in alignment with service delivery protocols. In the event of a reactive result, participants would have been referred for an urgent blood test at a specialist centre, and referral for treatment if the positive result was confirmed. All treatment for HIV is provided free of charge in the UK regardless of an individual’s personal circumstances. In these workplace events, there were no reactive results from the HIV tests. Twenty volunteers delivered the health checks, attending between 1 and 6 events (with 2–3 volunteers present at each event). HIV testing was provided by 8 sexual health professionals from one National Health Service (NHS) Trust and one charitable organisation, each delivering testing at between 1 and 10 events taking place between August 2019 and February 2020.

### Study design, participants, and data collection

This was a qualitative study using free-text responses gathered from 107 exit questionnaires and 24 qualitative interviews. Brief exit questionnaires were completed at the end of each event by all delivery partners, including health check volunteers (‘volunteers’) and sexual health professionals (‘HIV professionals’). The questions asked were, “what went well at the event?” and “were there any barriers to the success of the event?”

Eleven in-depth semi-structured interviews were conducted with volunteers and HIV professionals. These took place several months after the last Test@Work event, due to a period of national lockdowns occurring after the outbreak of the global coronavirus (COVID-19) pandemic in early 2020. Interviews were conducted remotely by phone or video call, with questioning informed by a topic guide (Supplementary file 1). Discussions explored interviewees’ experiences of conducting the events, factors impacting on the success of events and their views about including HIV testing in workplace events.

Semi-structured interviews were conducted with all 13 key contacts (‘managers’) who had organised the events at construction companies (some had organised multiple events). Interviews were either face-to-face immediately after an event or via telephone shortly after the event and followed a topic guide (Supplementary file 1). Questions were asked about interviewees’ experiences of the events, their views on the suitability of the workplace for conducting health checks and HIV testing, and their views on future health promotion activities (general and HIV-related) in their organisation.

**Fig. 3:**
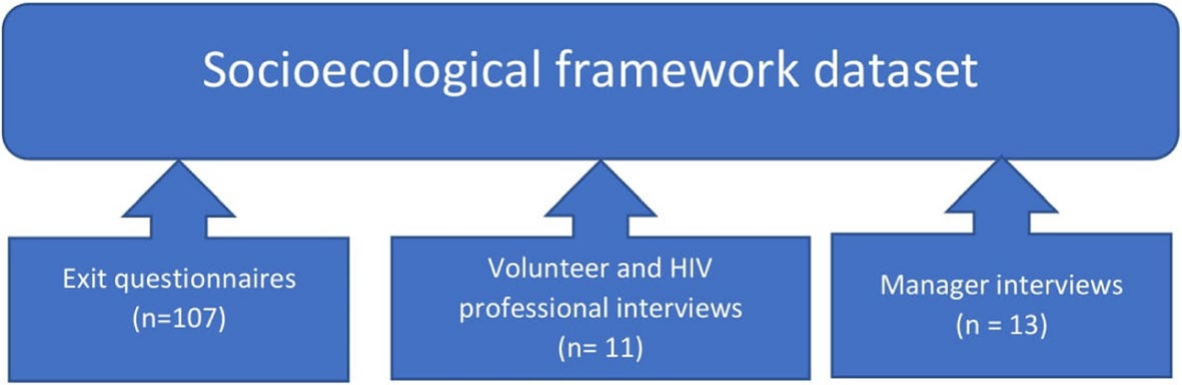
How the datasets were combined for use with the socio-ecological framework

All interviews were conducted by the same researcher, were audio-recorded with consent and fully transcribed. Three datasets were combined for use with the SEF in this study (Fig. 3). The study had approval from an institutional Research Ethics Committee (Ref: FMHS LT12042016) and the intervention was pre-registered (ClinicalTrials.gov Identifier: NCT04292002) and is described elsewhere [27–31].

### Data analysis

Thematic analysis was carried out using NVivo software (v12). Data sources were analysed as three separate datasets: i) free-text responses from volunteer/HIV professional questionnaires; ii) volunteer/HIV professional interviews; iii) manager interviews. In each case, a researcher read the transcripts several times and coded inductively, identifying the thoughts being expressed in the data [48, 49]. Coding was reviewed by a second researcher and revised where appropriate. The coded data were mapped against the SEF by two researchers working together. Finally, the three data sets were combined, and the coding structure reviewed and refined by two researchers working together to develop a single list of themes and subthemes within the framework. Any codes that did not fit within the framework were reviewed and revised by both researchers. This was followed by further review to ensure consistency and completeness of coding.

## Results

### Participant/data characteristics

In total, 107 questionnaires were completed by those involved in the delivery of events (*n* = 107, 100% completion rate). Of these, 37 forms were completed by 8 HIV professionals, 44 by 20 volunteers and the remainder by Test@Work programme team members (event organisers and clinical mentors supporting the volunteers). Volunteer and HIV professionals’ demographics are detailed in Table 1.

**Table 1:** Volunteer and HIV professional demographics (questionnaire participants)

Volunteers	*N* = 20
Gender	
Male	9 (45%)
Female	11 (55%)
Ethnicity	
Black and minority ethnic groups	13 (65%)
Other	7 (35%)
HIV professionals	*N* = 8
Gender	
Male	2 (25%)
Female	6 (75%)
Ethnicity	
Black and minority ethnic groups	5 (63%)
Other	3 (37%)

Interviewees were 7 volunteers, 4 HIV professionals, and 13 managers, see Table 2. Volunteers and HIV professionals who were not interviewed either declined an interview due to work commitments (*n* = 3) or did not respond to invitation to interview (*n* = 14). Those who did not respond were mostly individuals who had participated in only one event; others had reportedly changed jobs or left the university.

**Table 2:** Volunteer, HIV professional and manager demographics (interview participants)

Volunteers	*N* = 7
Gender	
Male	3 (43%)
Female	4 (57%)
Ethnicity	
Black and minority ethnic groups	4 (57%)
Other	3 (43%)
HIV professionals	*N* = 4
Gender	
Male	2 (50%)
Female	2 (50%)
Ethnicity	
Black and minority ethnic groups	2 (50%)
Other	2 (50%)
Managers	*N* = 13
Gender	
Male	10 (77%)
Female	3 (23%)
Ethnicity	
Black and minority ethnic groups	0 (0%)
Other	13 (100%)

### Research findings

The findings presented here identify characteristics of the construction sector and its employees which influenced perceptions and experiences of HIV testing, as seen by their managers and by health professionals providing testing. The emerging themes (summarised in Table 3) are mapped against a socio-ecological framework to guide future interventions. ‘Employees’ in this context refers to anyone associated with the organisation and working on the construction site on the day of the health check event, including employed, self-employed and agency workers.

**Table 3:** Summary of main themes, mapped against the SEF

Individual	Interpersonal	Organisation	Industry	Public Health
Male majority and masculine stereotypes Employee willingness to engage Mental health Perception of health risk Age, ethnicity	Peer effect on participation in health checks Employees sharing their knowledge with family and friends	Company engagement Site organisation, advertising, committed manager, booking Timing Location	Nature of the construction industry	Normalising HIV testing Stigma Novel approach to HIV testing: workplace HIV and HIV testing HIV education Opportunity to educate

### Individual factors

The construction workforce is predominantly male, and this impacts health-seeking behaviour. Interviewees from all groups commented on the reluctance of men to seek healthcare, in particular their reluctance to discuss sensitive issues,

*‘I think yeah, because the industry is predominantly male that might be the case of “ah, you know, just keep going, it’s fine, it’s fine.” (Manager)*

*‘Men don’t tend to see healthcare professionals until there is something really quite wrong and quite often leave things late.’ (HIV professional)*

However, strong engagement from men in HIV testing was evident within this study, and volunteers and HIV professionals identified the participation and engagement of the construction workforce as a key contributor to the success of events. HIV professionals reported that participants were open to discussing their sexual health and wellbeing in the workplace as a health promotion setting,

*‘They were actually really forthcoming with information … and they didn’t have any problems talking about sexual health in any way.’ (HIV professional)*

Several health check volunteers commented on employees’ willingness to discuss their mental health and often were surprised by this,

*“Men don’t talk about their feelings, men don’t talk about their mental health” … [but] there was some participants that came in and then they just told us like all what was going on in their life.’ (Volunteer)*

Mental health associated with masculine stereotypes was discussed by interviewees. There was recognition of the link between mental and physical health. Observations by HIV professionals highlighted that poor mental health could impact on HIV risk,

*‘Some people will go out and use sex as a self-harm … they will just go out and have random sex with random strangers … they are mentally harming themselves but also physically putting themselves in danger of catching things.’ (HIV professional)*

Despite the positive engagement of many with health checks, there were some employees who did not wish to participate in the events. Reported reasons for non-participation included a lack of time due to work demands, or having already had health checks elsewhere e.g., to gain work certificates for their jobs or feeling that it was not relevant to them. Some managers suggested that inclusion of an optional HIV test within the health check might have discouraged some employees from attending. Some HIV professionals and volunteers suggested that increased age, lower socio-economic status or being from an ethnic minority may reduce willingness to attend health checks but there was no clear agreement regarding this. For example, some felt older workers were less likely to attend than their younger counterparts, while others felt that older workers were more motivated to protect their health. One commented that,

*‘Like working class men, they grow up … you have a pain, tough, you know, you work through it.’ (HIV professional)*

but another reported,

*‘You might have older people that are perhaps a bit wiser and think “well, go for a test anyway” and perhaps take a bit more responsibility and as you get older you tend to realise your great health isn’t going to be great health forever.’ (HIV professional)*

One event was paused for a fire drill and an HIV professional noted dissimilarities between the wider workforce and those attending for health checks. They observed that there were many younger employees and black employees who may have had potential for HIV risk (e.g., from groups identified as ‘high-risk’) but did not attend. The interviewee was also concerned that knowledge about HIV was relatively poor amongst younger workers. Individual perception of health risk was a motivator for attendance at the event, this is particularly relevant in terms of HIV testing. Employees were observed by HIV professionals to be unaware of their risky behaviours including prior sexual partners (before entering a long-term relationship) and drug use. Others reportedly failed to understand the impact of changed circumstances on sexual behaviours and therefore HIV risk, such as becoming widowed or divorced, or having multiple sexual partners in later life without considering the risk their behaviour posed,

*‘Older people that either become unfaithful or get divorces, split up, or whatever, start having sex but haven’t got the information perhaps that a different demographic have kind of had access to, because they haven’t really had to think about it before.’ (HIV professional)*

For some accessing HIV testing, recreational drug use may have influenced their motivation to participate, ‘ *… [they] were sleeping with people at the weekends, having unprotected sex using cocaine … ’ (HIV professional).* Others were reported as having a history of marijuana use or having been in a past relationship with an intravenous (IV) drug user, although none admitted IV drug use themselves.

In summary, being male was perceived to reduce the likelihood of construction employees seeking health care. However, once engaged in health checks, they were enthusiastic and interested in health promotion and sexual health awareness. Nonetheless, many had poor understanding of their own exposures to HIV risk.

### Interpersonal factors

Peer pressure, both positive and negative, was reported as an influence on participation in health checks. Volunteers and managers reported that employees encouraged their colleagues to participate in health checks once they had taken part themselves,

*‘I think it was more the fact that everyone else was doing it, so I think it was … because all of their colleagues were getting it done, it was like “oh, I guess I will go and get that done now then.” ’ (Volunteer)*

However, peer pressure could also be a negative factor, with some raising concerns that employees may feel coerced into participating by the enthusiasm of their colleagues,

*‘They were all you know sort of egging each other on but also, I think that that could also … ..have had the opposite effect as it’s a place of work they … may have felt a little bit sort of self-conscious with the health check.’ (Volunteer)*

The potential for knowledge sharing amongst employees’ friends and families was identified, with a ‘trickle’ effect for raising awareness of HIV and reducing stigma surrounding it,

*‘ … challenging the stigma and also educating individuals who may not necessarily go out of their way to access that kind of information … sharing that amongst their colleagues but also much wider within their own communities and own families.’ (HIV professional)*

*‘I mean, even just when you’re having a chat with your partner at home about it. You know, word gets round with that sort of stuff.’(Manager)*

In summary, peer pressure can influence an individual’s health-seeking behaviour, including accessing opt-in HIV testing. Knowledge sharing about HIV outside the event was seen as a positive outcome which could increase the impact of HIV awareness raising beyond those being tested at events.

### Organisation factors

The research showed that a high level of company engagement was critical to the success of the health checks. Good marketing and event promotion encouraged employees to attend, and ensured they knew what to expect, including the fact that HIV testing was part of the event,

*‘There appeared to be a bit of a misunderstanding of the people that were coming to the sessions and exactly what was expected of them or what we were doing, and I think when they realised, I think they found it more helpful than they expected to.’ (Volunteer)*

An associated factor was the time that companies invested in planning events and facilitating the pre-booking of health check appointments. Where this was managed well, there was a good flow of employees, and events flowed smoothly; where it was not, there was often a flurry of employees followed by quieter periods, and staff sometimes felt under time pressure with health checks. Having a motivated manager on site to help run events minimised these problems. This happened on the sites which appeared to be the most organised internally and had good communication, which yielded high participation rates and good employee engagement,

*‘He was proactive, he was going out there getting people to come out … he went into offices and then he created slots for people to come and he gave them kind of short ten-minute breaks to come and meet us.’ (Volunteer)*

On sites where events ran most smoothly, planning and communications took place in advance of the event, and the site manager was present to support the delivery team throughout the day. At less successful events, by comparison, there was no named contact person for the delivery team on site, no-one to liaise with about appointments and events sometimes started late due to difficulty gaining access to suitable rooms. For example, at some sites the teams were relocated part way through events due to companies’ lack of understanding of space requirements or time schedules for these events. This was often because the pre-event information had not been appropriately reviewed by the companies or sites involved.

The quality of facilities provided for health checks and HIV tests also influenced how they were experienced. Practical challenges for HIV professionals and volunteers included rooms which were too small, poorly located (e.g., remote from the main site, close to public areas, or split between two rooms with a long distance between them), or offered limited privacy,

*‘You would be plonked in a room that was quite unprepared or even dirty at times, I think one of the rooms smelt of petrol, that they were going to do the HIV checks in, or they didn’t realise that we needed more than one room.’ (Volunteer)*

There were concerns around ensuring employee privacy during some health check events, this was influenced by the quality and location of rooms provided and volunteers and partners had to be adaptable to find ways to work around this,

*‘Having, that private space for HIV testing was really important … talking about something as private as somebody’s sex life or personal life in a public space wouldn’t have been beneficial to the participant and wouldn’t have been beneficial for us if we were to deliver a reactive result.’ (HIV professional)*

Almost all of the comments about limited or poor space came from the volunteers or HIV professionals. The managers organising events on site very rarely mentioned this as a factor which had limited the success of the event.

In summary, company engagement impacted on the success of the health check events and the suitability of the location offered for opt-in HIV testing and health checks. The practicalities of offering health checks and HIV testing on construction sites can be complex and consideration of location and managerial support on site is important. Volunteers and HIV professionals identified issues with location and room suitability, but this was not noted by managers.

### Industry factors

The underlying structure and organisation of the construction industry introduced challenges to the delivery of health check events, but also meant they were particularly valuable for employees attending. One factor mentioned frequently was the importance of healthcare being convenient and accessible for this workforce, as they are unlikely to take time out to attend otherwise. Employees may be living away from home or may have long working hours, long commutes, and little free time. They will therefore not leave site for health checks, particularly as they would lose income if they did so,

*‘They’ll be in at 7 and then they’ll be gone at 3 … some of them probably travelled a couple of hours to get here and … because a lot of them get paid for the amount they do, by going to the doctor’s they’d probably lose a whole day’s money.’ (Manager)*

Even with events organised on site, some employees declined attendance due to the associated financial costs. This appeared to be more prevalent in those who were self-employed or subcontracting and was less of a barrier to those employed by the main contractor who could attend in company-paid time,

*‘The nature of the work out there, some people … didn’t want to take a minute out … because they’re on price work, they want to keep working all the way through, which is fair enough.’ (Manager)*

Additionally, some declined health checks due to high work demands and tight programme deadlines. These factors all reflect the way the UK construction sector operates. One manager pointed out how difficult it was to recruit employees to attend health check events because of the multi-level nature of the business,

*‘And the trouble is as well because of the way we work here, all the subcontractors are employers in essence; so, I’m employing them … but they’re employing another team … They employ the subcontractor that employs a ‘ganger’ that probably bring four or five people of their own with them, so … there can be several employers involved if you know what I mean.’ (Manager)*

Further issues arose from this complex organisation. For example, some employees were already getting access to health checks from their own employer and gave this as a reason to not participate in the event. Conversely, others were offered nothing by their (often small) employer so the health check events were welcome. The simple health check provided was perceived very positively, but some managers suggested it did not fully meet the needs of all employees and that other work-specific health checks could be provided, such as lung function testing or hearing tests.

The workforce was reported to be highly transient, often moving between different sites to complete different tasks. It would therefore be difficult to offer health checks to everyone connected with a particular site unless multiple events were organised. Despite this fragmented workforce, there appeared to be a strong motivation amongst participating companies to support and improve the health of workers. This was perhaps motivated by the poor lifestyle choices which some interviewees identified were evident in the construction workforce,

*‘They said … “we’re up in the morning and we get as much sugar" … the majority of these people live on red bull, chocolate bars and sugar to keep them going.’ (HIV professional)*

All managers interviewed gave examples of current interventions from their companies which targeted employee health, including promotions to encourage exercise; offering opportunities for health checks and health screening; and providing a range of support and education in the field of mental health. The health check events were therefore frequently part of a wider programme of health improvement, often driven by larger, parent companies. Some managers were committed to this agenda for either moral or business reasons. Some spoke of the need to improve the longer-term health and effectiveness of workers, while others were doing it to achieve accreditations or to be recognised as a good employer,

*‘We are a lot more focused on this sort of thing nowadays … we’re being asked to sort of try and promote and push things like this as well so, yeah we’ve gotta learn about it.’ (Manager)*

Events were particularly valued because there was no direct cost involved to the companies or sites taking part. Several managers expressed interest in arranging additional events after the study end. However, despite the high motivation, there was limited confidence that companies would take HIV testing or education forward without outside involvement. This was because a decision would need to be made at a more senior level in the parent companies, as part of the agenda for the whole business and this did not currently include sexual health awareness,

*‘Our health and wellbeing programme is run centrally by HR … I can certainly feed in information to our regional HR manager, but whether that would get incorporated … I honestly don’t know.’ (Manager)*

In summary, construction has a fragmented and transient workforce which limits opportunities for employees to seek health care. The companies participating in this research were generally keen to engage in interventions which might improve worker health, but there was limited scope for individual sites and projects to initiate health-promoting activities unless they were driven by parent companies, particularly if there were associated costs.

### Public health factors

HIV testing in construction workplaces can contribute to the wider public health agenda. A strong theme discussed in interviews was normalisation of HIV testing. Opt-in HIV testing, within the context of a general health check, was highly acceptable to employers and employees and led to high testing uptake. Interviewees believed that fewer employees would have accessed HIV testing if it was offered in isolation, and even fewer if attendance at a sexual health clinic was required. This reflected the stigma that is seen to still attach to the disease. Employees were willing to be tested in this environment because it did not require any admission of being at risk, it was part of what one HIV professional referred to as a *‘conveyor belt’* effect where employees came for health checks and took part in everything on offer. It was therefore a non-stigmatising opportunity to access the test,

*‘It’s a lot easier to sell and describe to people: “what have you done at work today?” “Oh, I’ve had an HIV test” or “what have you done at work?”, “Oh, I’ve had a health check.’ (HIV professional)*

At the same time, it created an opportunity to lessen the stigma because of its position with other, more familiar tests,

*‘I think having it as a routine thing is excellent because it takes away some of that fear and stigma because everybody says … “this is what normally happens and this is what everybody does.” ’ (Volunteer)*

Employee experiences of testing helped to further mitigate perceived stigma. Those who did not initially think the test was relevant to them valued the opportunity to discuss their sexual health and recognised the benefit of being tested. It was also recognised by some managers that offering testing was important, given that HIV was now a condition which is manageable rather than life-limiting,

*‘The bit on HIV puts people’s mind to rest around it being a managed condition as opposed to something that cannot be managed and the life expectancy from when it all kicked off back in the 80’s … what you’re doing is no different to having a blood test for diabetes or the rest so, you know, both are manageable.’ (Manager)*

The benefit of introducing HIV testing into novel community environments (e.g., workplaces) was highlighted by the normalisation seen here as many individuals accessed testing who would have been missed via traditional routes (e.g., healthcare services, sexual health clinics). All HIV professionals commented on the extent to which employees had tested ‘*so many different people who wouldn’t necessarily come into a clinic’*. One commented that the workplace was a good opportunity to access an under-served population, who would have struggled to attend specialised centres due to practicalities including work commitments. Another HIV professional indicated that they would rarely test so many heterosexual men in their clinic environments, and that,

*‘When you have got a population that you think are less likely to be at risk or have HIV, it can also be some of those people that don’t come forward for testing so therefore are missed and end up with a late diagnosis.’ (HIV professional)*

The actual experience of providing HIV testing in the workplace setting was different for HIV professionals compared to their usual experiences in clinical settings. They commented that those who attended for testing in traditional settings would have made an appointment, and a conscious decision to discuss their sexual health, whereas those in the workplace were sometimes less willing to ‘open up’, either because it was an unfamiliar situation for them, or because they had concerns about confidentiality.

In particular, interviewees identified the challenges that would have arisen should employees be given a reactive result; the importance of a suitably private location for this, the unexpectedness for employees who might not have recognised their risk,

*‘At least if you go to somewhere like a sexual health clinic … you are expecting that, you know, you are prepared for either outcome because you are seeking out that service yourself. But if I went to work one day and got told I had HIV … it is quite blind-siding isn’t it?’ (Volunteer)*

The HIV specialists discussed their experiences of testing on a construction site compared to their usual location. In many ways, their approach was the same, focusing on the importance of removing barriers to encourage engagement with the services they offered. They had to adapt to the fact that those accessing testing may never have been offered an opt-in HIV test before or been educated regarding HIV and sexual health, and some seeking testing may be less at-risk than the high-risk populations they would routinely see in clinics,

*‘I think in terms of the discussions and the conversations, we had to tweak it because … we are all about testing the right people. So, if somebody poses that they have not had any risk we wouldn’t test them because it is a waste of resources and it is a waste of discussions … but you know for people who had never tested before, it was also probably a good experience because they have never had a HIV test.’ (HIV professional)*

They also had to adjust their discussions to work in a shorter, more constrained time slot than they might in their usual clinic environment and had to take a narrower approach due to the nature of the Test@Work programme. This included focusing primarily on HIV where usually tests would have also been offered for other sexually transmitted diseases (e.g., chlamydia). A testing opportunity may have been missed here, given some employees specifically asked about a bank of tests for sexual health,

*‘if you can do a more general sexual health check that would probably be beneficial for a lot of the lads because they were asking “ooh is it just HIV or do they do other ones as well.”’ (Manager)*

*‘I think if we could have tested for, well, particularly gonorrhoea and chlamydia, I think we definitely would have got some positives from that, and there was a lot of men that we talked to that just had never been tested for anything but were clearly at risk for some of them.’ (HIV professional)*

The use of a finger prick test was mentioned as being more acceptable than a venous blood sample to those being tested. This point-of-care test involved taking a small spot of blood from the individual’s finger, the sample did not need to be sent to a laboratory and the result was available within a few minutes. One HIV professional commented on the benefits and limitations of point-of-care testing, that it allowed access to an untested population but introduced challenges of cost and accuracy, with reactive tests requiring follow-up testing. Another observed that postal testing was available in some geographical areas but not in others which may introduce health inequalities.

The value of the workplace as a route for HIV education was discussed. Firstly, it was important for those attending for testing to have sufficient information in advance so that they knew what to expect and could make an informed judgement about testing. More broadly, education was seen as being very important to combat poor understanding of risk factors for infection. Other important messages identified were that once treated, the disease is manageable, so that it is ‘*not the end of the world’* and that effective treatment eliminates the risk of spreading the disease. Improving education was viewed as a mode for stigma reduction relating to HIV and to improve socially responsible behaviour and reduce disease spread.

In summary, workplace opt-in HIV testing, when embedded within a package of general health checks, is viewed positively and is a useful mechanism for normalising HIV testing and reducing HIV-related stigma. This approach is useful for reaching populations that might not independently access healthcare services, but the type of test used needs to be further considered. There is scope for workplaces to provide a platform for a wider repertoire of health tests for construction workers.

## Discussion

This is the first study to explore the perceptions of managers and delivery partners towards workplace HIV testing in the construction workforce. We found that opt-in HIV testing, when delivered within the context of a general health check, is an effective way to target a population who would not otherwise seek testing. It therefore has the potential to increase early HIV diagnosis, which is a public health priority [8] to reduce morbidity and disease spread.

### The nature of the construction workforce

Construction has a largely male population. The reluctance of men to seek healthcare, as discussed in this paper, is recognised in the wider literature [50, 51]. In the construction industry specifically, there are practical barriers which prevent employees seeking healthcare such as lack of free time and negative financial consequences [21, 32]. The high level of engagement of construction employees in workplace health checks (a setting where convenience is maximised, and financial impacts minimised) shows that practical barriers to testing are at least as important as the stereotypes related to masculinity. This aligns with the conclusions reached by Tyers and colleagues [52] who identified that “individuals within the sector are interested in their own health and taking steps to protect it, despite what employers might think”. It also highlights the benefits of taking public health intervention directly to the workforce in this way to overcome barriers, whatever the cause.

The challenges of improving health in the construction workforce are widely recognised in construction [36, 53, 54]. In particular, there have been substantial efforts [55, 56] to address the mental health issues prevalent in the population which are reflected in its high suicide rate [57–59]. Poor mental health can increase HIV risk – not just via the risk of self-destructive sexual behaviours, as identified in this study, but also the potential for increased drug and alcohol use which is a recognised negative coping strategy in the sector [60, 61] and which can independently increase HIV risk [25].

### The nature of the construction industry

The construction companies which participated in this study were enthusiastic about offering health checks, both to address general health and HIV specifically. However, a range of practical and logistical challenges arose, due to the complex structure of the sector, the transient nature of many employees and the poor facilities on many sites. Poor managerial engagement and failure to provide the support required for events to run smoothly was also an issue in some organisations. These factors have been previously acknowledged as barriers to the provision of good health management in construction. For example, a large HSE (Health and Safety Executive in the UK)-funded project to support construction companies found that the reliance on subcontracting arrangements, the short-term nature of projects and the reluctance of many employers to take ownership for the health of their workforce all introduced challenges [52]. The fact that construction projects run on very low profit margins, and often struggle to meet deadlines [62] are likely to be additional factors which discourage employers from committing resources to employee health and wellbeing. Poor facilities are also a known issue: many construction sites struggle to even provide adequate welfare facilities for their workforce [63], and a poor work environment and lack of cleanliness have been identified as stressors for construction managers [64].

Where health checks including opt-in HIV tests are to be offered in construction it is essential that very clear guidance is given to all parties regarding minimum standards needed for safe, private HIV testing. Given that not all sites will be able to offer suitable facilities, approaches may need to be adapted to the setting. For example, occupational healthcare in construction is often provided through the use of mobile facilities [52, 65] and this is an approach which could be considered.

### Models of HIV testing

This research sought to provide HIV testing to a previously untested population, through the construction workplace setting. Interviewees observed that many employees had been unaware prior to the event that they were at risk, but also noted that the risk exposure of the overall population was lower than they might see in sexual health clinics. This meant the likelihood of a positive diagnosis was lower than in other settings; no tests produced a reactive result in this study. Importantly, employees may be less prepared to receive a reactive result, compared to individuals attending an HIV clinic, who have primed themselves mentally for testing and are likely to have a good understanding of their degree of risk exposure.

This research used rapid diagnostic testing, which gave employees their results within 15–20 minutes of testing, and this was highly acceptable to employees [28]. Rapid testing has previously been reported as being acceptable to clients and as increasing the probability of individuals receiving their results compared to other testing strategies [3]. It is the testing approach recommended by the WHO for community settings, as it is easy to perform and ensures good linkage to follow up care and treatment [66]. However, delivery partners in this study expressed concerns regarding the potential for challenges in delivering reactive results sensitively and confidentially in some construction environments, so other approaches should be considered. Examples of these are reported in the literature. For example, employers have offered vouchers to allow testing offsite [24]. Opportunistic testing in clinical settings (ie., during routine or emergency healthcare) has used oral fluid sampling, venous samples or dried blood spot (DBS) testing, with results later discussed with the client by health professionals by phone or face-to-face [10]. Access to self-test kits with follow up support is also a successful strategy [67, 68]. Dried blood spot testing, for example, involves a finger prick test with a sample collected onto specialised filter paper then sent for laboratory analysis. DBS also offers scope for wider testing such as the combined testing of HIV, Hepatitis B and Hepatitis C [69]. Such an integrative approach for blood borne viruses is advocated to reduce onward transmission, particularly considering the similarity in risk factors, the prolonged asymptomatic period and the importance of early diagnosis and treatment [70]. Such approaches could therefore be explored for workplace use as an alternative to rapid diagnostic testing, given that results are not given at the point of care which may be preferable where onsite facilities are poor. Remote services for HIV prevention and treatment have developed rapidly, because of the COVID-19 pandemic [71, 72], and this might also feed into new models for the provision of accessible HIV testing in workplaces.

This study sought to normalise HIV testing by providing it alongside other health checks in a workplace environment. This was an effective way to access those who might not be tested otherwise, particularly those who may be at-risk, but not recognise this. This approach is less discriminatory than targeted testing based on risk factors such as ethnicity or country of origin [1]. Offering HIV testing to all (‘universal offer’), regardless of perceived risk is a valid approach to HIV screening. In the United States of America (USA), testing is recommended for all adults [73] and in the UK and much of Europe, testing is offered to all pregnant women [11]. Youssef et al. [74] have advocated routine screening for the over 50s in the UK given that clinicians often underestimate HIV risk in this population. A more common approach is for HIV programmes to ‘*aim to reach those at risk of infection and to prioritise those at highest risk’* [8]. Testing in the UK is largely offered based on local HIV prevalence and patient risk [2]. This targeted approach minimises the increased costs which are associated with a low case diagnosis rate [19]. The high cost of testing and of case finding is therefore a potential barrier to more widespread and non-targeted testing in workplaces. The cost of workplace testing would be further increased if additional tests were included such as hepatitis B and hepatitis C (e.g., Matulionytė and colleagues [75]). Conversely, including these tests might increase the number of cases of blood borne viruses identified overall; and the cost of case finding for hepatitis B and hepatitis C is often lower than that for HIV [76, 77] therefore improving the overall cost/ benefit balance.

There might also be scope for compromise in work-based testing by targeting construction projects which have a greater proportion of higher risk employees (e.g., those in London, UK which are more ethnically diverse and also have more employees who are living away from their families [62, 78, 79]). Improving coverage of employees on sites tested might also be of value. At some sites, a discrepancy was observed in this study between those attending for testing and the overall site population, and this may reflect reluctance to be tested due to fear of a positive result amongst those at higher risk [80, 81].

### Education

The need for improved education and awareness-raising regarding HIV was highlighted by this research. Older construction employees were often seen to underestimate the risks arising from their past sexual activities, while younger people were seen as being at risk due to their low awareness of the disease. This lack of knowledge has been reported elsewhere, e.g., the over 50’s in the USA have been reported to have poor understanding about HIV transmission [82] and to engage in risky behaviours [83]; and adults in Japan have varying knowledge about HIV risk factors [84].

Where HIV testing is to be provided in workplaces, education is important to ensure that employees are well-informed about what to expect, so that a decision to participate is properly informed [8] and not made under pressure [12]. It would also be beneficial to provide educational resources in construction workplaces more widely, given the recognised existence of risk factors [25, 26], and that HIV rates are typically higher in populations with lower qualification levels [85]. Improved knowledge about HIV has been associated with a reduction in risky behaviours [86]; with increased HIV testing in the over 50s [74] and with reduced stigma [13, 84, 87]. It may encourage those who are at risk to seek testing, so that cases can be found even if widespread onsite testing in the sector is not achievable.

The construction companies that participated in this research were all engaged in health promotion activities to some extent, and the managers interviewed took the health of their workforce seriously. All of the participating worksites received a digital training package [29] prior to the Test@Work health check events which was evaluated positively by managers. The package provided general information about workplace health screening and HIV testing (what to expect, processes), and included detail about the roles and responsibilities of employers should an employee declare that they were HIV positive. However, for many managers in our study, distributing further education about HIV to their employees (beyond that required for the research study) was not a priority, as health agendas were set by parent companies and did not include sexual health awareness. This aligns with previous research showing that opt-in HIV testing is exceptionally rare in UK organisations, despite positive attitudes of employers towards it [88]. Embedding HIV in workplace health education programmes would therefore be best achieved by directly targeting the largest employers or projects, who set agendas for which aligned (often smaller) organisations adhere. Ensuring suitable resources are available for employers to use would also be helpful. Such materials are often produced to support workplace health promotion (e.g. [89]), but typically focus on cardiovascular and mental health risks rather than sexual health.

### Strengths and limitations

This study provides novel insights for opt-in HIV testing as a component of workplace health promotion. Although the dataset was relatively small it was based on detailed interviews with highly knowledgeable individuals. For example, the HIV professionals were vastly experienced in delivery of community-based testing. All attended multiple Test@Work events and were equipped with a good understanding of the project and the challenges and impact that HIV testing within the workplace may have. These are all factors which increase information power [90]. Additionally, gathering data from all those delivering health checks and from construction managers enabled barriers and facilitators to be identified from an industry and a healthcare perspective. The brief questionnaire data from all volunteers and HIV professionals present at each event provided a larger sample, albeit with less detailed content, which could be used to confirm these findings. Some volunteers could not be reached for interview at the time of this study. Those not captured for interview generally attended only a single event and it is unlikely their responses would alter the overall conclusions of this study.

## Conclusions

The nature of the construction sector with its complex structure and employment relationships, and the transient nature of projects, reduces the likelihood of this population seeking healthcare due to the inconvenience and costs of taking time away from work. Additionally, this is a largely male population, who are often identified as being reluctant to seek healthcare. Workplace testing for HIV within the context of a general health check on construction sites allows access to a population who might not otherwise be tested. This approach aligns with recommendations to normalise testing, to encourage testing uptake and reduce HIV-related stigma. Construction workers also have poor knowledge about HIV risk which makes education and awareness-raising important, either in addition to testing provision or in isolation. Construction projects present practical challenges to onsite HIV testing, with some sites lacking adequate space and facilities, compromising the ability to ensure suitable privacy. Detailed planning and collaboration between each site and those offering testing is essential. Rapid testing may not be the best process to use in some construction environments and alternate methods of testing should be explored.

## Supplementary Information

**Additional file 1. **Interview topic guides.

## Data Availability

The datasets generated and/or analysed during the current study are not publicly available to protect the confidentiality of employees and industrial participants but are available from the corresponding author on reasonable request.

## Abbreviations

COVID-19: Coronavirus disease
CDC: Centers for Disease Control and Prevention
HIV: Human immunodeficiency virus
HSE: Health and Safety Executive
UK: United Kingdom
USA: United States of America
SEF: Socio-ecological framework
WHO: World Health Organization
