# Supplementary material for: Opt-in HIV testing in construction workplaces: an exploration of its suitability, using the socioecological framework
Source: BMC Public Health. 2022 Jul 23;22:1409. doi: 10.1186/s12889-022-13787-5 (PMC9308504; doi:10.1186/s12889-022-13787-5)
Supplement: Supplementary file 1 — Additional file 1. Interview topic guides. [file 12889_2022_13787_MOESM1_ESM.pdf]

Additional file 1. Interview topic guides

Topic Guide 1: Volunteers - Post Project Interview

What was it like for you being involved in this project and why did you volunteer for it? What was the experience like working in an industry setting with other health professions?

Working with other students / senior healthcare professionals

- How did you benefit from this? Did you face any challenges?
- Have you developed personally through participation?

Working as a team / taking responsibility / helping others / interacting with different professions

- Do you feel your skills were developed prior to and during these events or if you helped to develop others you were working with? If so, how? If not, why?
- Would you personally do anything different in the future? If so, what?
- Was any additional support needed?

Conducting checks at the events / organisation / personally in terms of development

- Did you encounter any surprises or impact you were not expecting along the way?
- Learning in a real-world environment / impact of this?
- Would you do it again? If yes, why / If no, why

Have you volunteered for anything else (or similar) since your involvement with this project?

What do you remember about things that went well, or that helped event(s) run smoothly?

- *Prompts* engagement of company, employees, checks being free, location, timing, what were the things that happened at the best events?

Do you remember anything challenging or tricky about any of the events?

- *Prompts* any organisational issues, location etc. Did anything go wrong? Location, timings, engagement of management

What are your views on including HIV testing as part of a general health check?

- *Prompts*: Good / bad, what appeared to drive people to access or not access testing?

How do you think people felt about being offered testing in their place of work?

- What do you think were the main reasons why people took part or not?
- What are your own views on including HIV testing in a workplace health check?

How do you feel about the Test@Work project overall?

- In your view, what could have been better?
- What would you suggest we do differently if we did it again?
- What is the key thing you will take away from these events? Personally? Professionally?

Topic Guide 2: HIV Professionals - Post Project Interview

What are your views on including HIV testing in workplace health checks?

- *Prompts*: Good / bad, what appeared to drive people to access or not access testing? How do you think people felt about being offered testing in their place of work? What do you think were the main reasons why people took part or not?
- *Prompts*: What factors could encourage engagement?

What was your experience of delivering HIV testing in the workplace health checks?

- *Prompts*: what was covered with those participating in the discussion prior to the HIV test being offered?
- *Prompts*: What would the process be for a reactive test result?

In your view, what were the key factors that facilitated the events?

- *Prompts:* engagement of company, employees, checks being free, location, and timing, what were the things that happened at the best events?

In your view, what was challenging about the events?

- *Prompts*: any organisational issues, venue etc. Did anything go wrong?
- *Prompts*: how does this differ to the way you would usually test? Does it offer any additional benefits?
- *Prompts*: location, timings, engagement of management

What are your opinions of the online health testing toolkit? And your views on provision of this to employers?

How do you feel about the Test@Work project overall and what was it like for your organisation to be involved in this project?

- What have been the benefits? Were there any problems? Did you encounter any surprises or impact you were not expecting along the way? Would you do it again?
- In your view, what could have been better? What would you recommend in the future?

Topic Guide 3: Managers - Post-Event Interview

Have you offered health promotion activities at your organisation before? If so, what sort of activities?

Tell us about your views and experiences of the health check events

In your view, what are the factors that influenced attendance at the health events?

- *Prompts*: Internal advertising/promotion? Timing? Other? What might have hindered attendance at the events?

Can you tell us your views on the content and focus of these events?

How do you feel about including the provision for HIV testing in workplace health checks?

Can you tell us what you thought of the digital toolkit?

- *Prompts*: Was it useful? In what way? Would you refer to it in the future? Would you recommend it to other organisations? Any other comments about the toolkit?

Can you tell us whether the events and/or the toolkit have influenced the company's future health and wellbeing strategies or activities in any way?

Can you tell us whether the events and/or the toolkit have influenced the company's future view on HIV awareness and testing in any way?
